# Improving Visible Light-Absorptivity and Photoelectric Conversion Efficiency of a TiO_2_ Nanotube Anode Film by Sensitization with Bi_2_O_3_ Nanoparticles

**DOI:** 10.3390/nano7050104

**Published:** 2017-05-09

**Authors:** Menglei Chang, Huawen Hu, Yuyuan Zhang, Dongchu Chen, Liangpeng Wu, Xinjun Li

**Affiliations:** 1 College of Materials Science and Energy Engineering, Foshan University, Foshan 528000, Guangdong, China; mengleic@sina.com (M.C.); mengleic@sina.com (H.H.); cdcever@163.com (D.C.); 2 Key Laboratory of Renewable Energy, Chinese Academy of Sciences, Guangzhou 510640, Guangdong, China; wulp@ms.giec.ac.cn

**Keywords:** Bi_2_O_3_ sensitized TiO_2_, photoelectric conversion efficiency, visible light-active, nanoparticles

## Abstract

This study presents a novel visible light-active TiO_2_ nanotube anode film by sensitization with Bi_2_O_3_ nanoparticles. The uniform incorporation of Bi_2_O_3_ contributes to largely enhancing the solar light absorption and photoelectric conversion efficiency of TiO_2_ nanotubes. Due to the energy level difference between Bi_2_O_3_ and TiO_2_, the built-in electric field is suggested to be formed in the Bi_2_O_3_ sensitized TiO_2_ hybrid, which effectively separates the photo-generated electron-hole pairs and hence improves the photocatalytic activity. It is also found that the photoelectric conversion efficiency of Bi_2_O_3_ sensitized TiO_2_ nanotubes is not in direct proportion with the content of the sensitizer, Bi_2_O_3_, which should be carefully controlled to realize excellent photoelectrical properties. With a narrower energy band gap relative to TiO_2_, the sensitizer Bi_2_O_3_ can efficiently harvest the solar energy to generate electrons and holes, while TiO_2_ collects and transports the charge carriers. The new-type visible light-sensitive photocatalyst presented in this paper will shed light on sensitizing many other wide-band-gap semiconductors for improving solar photocatalysis, and on understanding the visible light-driven photocatalysis through narrow-band-gap semiconductor coupling.

## 1. Introduction

Study of effective photocatalysts lies in following conditions: (i) the prepared photocatalysts are capable of harvesting the solar energy of full wavelength as much as possible; (ii) high photocatalysis efficiency [1,2]. Due to the odorless, non-toxic, and chemically stable properties of TiO_2_, it is widely employed and deeply analyzed for various kinds of applications [3,4,5,6,7,8,9,10]. However, TiO_2_-based photocatalysts are only able to capture the ultraviolet (UV) part of the solar light; the UV light energy is comprised of only 4% of the total solar energy reaching earth, along with a rather low photocatalysis efficiency being lower than 1%, in contrast to 43% when visible light region is concerned [11,12]. As a consequence, the current focus is placed on exploration of new visible light-active photocatalysts or modification of existing photocatalysts to extend the solar light absorption to the visible light region [13].

As one of most widespread applications of TiO_2_ materials, dye-sensitized solar cells (DSSC), involves the use of a nano-porous TiO_2_ film as semiconducting electrodes, with high specific surface area [14]. The DSSC is a photoelectrochemical solar cell, which employs organic compounds containing metallic Ru, Os, etc. as dye sensitizers and selects proper redox electrolytes. The photoelectrical efficiency of a DSSC was recently reported to achieve as higher than 11%, which can also arrive at 6% as far as large-scale applications are concerned [15,16,17,18]. Such efficiencies are much higher than that for un-sensitized photocatalysts. However, most organic dyes used in DSSC are unstable, causing instability of the DSSC [16]. To address this, stable narrow-band-gap semiconductors can be resorted and used as a sensitizer for visible light-activation of TiO_2_, replacing the organic dye in DSSC. Similar to the function obtained in DSSC, the semiconductor with a narrow band gap can efficiently harvest the solar energy, while wide-band-gap TiO_2_ is able to separate the photo-generated charge carriers.

The significance of using visible light-active photocatalysts to sensitize TiO_2_ is to improve solar light absorption of TiO_2_ [19]. Different energy band gaps between the sensitizer and TiO_2_ lead to the generation of a built-in electric field [20], causing the photo-excited charge carriers to inject from one semiconductor to the other [21]. This inhibits the recombination of the generated charge carriers, in the meantime, the photo-generated electrons and holes can be well separated, improving the photocatalytic activity [22]. As a result, such combined semiconductors always exhibit a higher photocatalytic activity than that of the single-component semiconductor [23]. In order to combine semiconductors with different energy band gaps, we need to consider (i) band gaps of these semiconductors; (ii) positions of the valence band (VB) and conduction band (CB); and (iii) the match of crystalline forms of these semiconductors. The methods and procedures used to prepare the combined semiconductors also have an influence on the final photocatalytic activity [24].

For the first time, this paper reports the sensitization of TiO_2_ nanotubes by coupling a visible light-active semiconductor Bi_2_O_3_, affording a good a visible light-absorptivity; this thus also indicates the enhancement of the solar light absorption. Selecting Bi_2_O_3_ for sensitization of TiO_2_ is due to (i) its narrow direct band gap of 2.8 eV; and (ii) suitable VB and CB positions for transporting and separating the photo-generated charge carriers. Because the photo-generated electrons and holes can be trapped and transported by these different semiconductors, both the photo-generated electrons and holes can be concentrated simultaneously, enhancing the oxidation/reduction capability of the electrodes in prepared solar cells. The photocatalytic and photoelectric conversion efficiencies are improved correspondingly. This paper presents the detailed synthesis of the visible light-active composite of Bi_2_O_3_-sensitized TiO_2_ nanotube film. The structure and photoelectrical properties of the composite are also well unraveled.

## 2. Experimental

### 2.1. Materials

Bismuth nitrate hydrate (Bi(NO_3_)_2_·5H_2_O) of AR grade was supplied by Guangzhou Chemical Company (Guangzhou, China). Tetra-*n*-butyl titanate (Ti(OC_4_H_9_)_4_) of AR grade was obtained from Xinhua Active Materials Institute (Changzhou, Jiangsu, China). Diethanol amine (NH(C_2_H_5_OH)_2_ ) of AR grade was purchased from Luoyang Chemical Reagent Factory (Luoyang, China). Polyethylene glycol (M_W_ 20,000) was provided by UNI-CHEM (Hong Kong, China), with AR purity. All other chemicals were of AR grade and used as received.

### 2.2. Synthesis of TiO_2_ Nanotubes

The synthesis of TiO_2_ nanotubes was based on a hydrothermal route. Specifically, 2.0 g of purchased TiO_2_ with anatase form was mixed into a NaOH solution (10 mol/L) by continuously stirring at 110 °C for 12 h, followed by cooling to room temperature. The white precipitate was then collected and thoroughly washed with deionized (DI) water until the electrical conductivity of the supernatant was lower than 70 μS/cm. The washing procedures were continued by dipping the precipitate into a HCl solution (0.1 mol/L) and letting it stand for 5 h, and then by further washing with DI water until the electrical conductivity of the supernatant was lower than 5 μS/cm. Afterwards, the washed TiO_2_ was vacuum-dried at 60 °C for 72 h.

### 2.3. Preparation of TiO_2_ Sol-Based Film

Using Ti(OC_4_H_9_)_4_ as a raw material and NH(C_2_H_5_OH)_2_ as an inhibitor to impede fast hydrolyzation of the titanate. The addition ratio of the Ti(OC_4_H_9_)_4_, EtOH, DI water and NH(C_2_H_5_OH)_2_ was kept at 1:26.6:1:1. A precise amount of Ti(OC_4_H_9_)_4_ was dissolved into ethyl alcohol (EtOH), along with addition of NH(C_2_H_5_OH)_2_. The mixture was agitated for 1 h, leading to liquid A. The DI water was homogenized with the remaining EtOH, forming liquid B which was then stepwise added into the liquid A through a separating funnel, followed by continuous stirring for 0.5 h and then letting it stand for 24 h. The resulting stable TiO_2_ sol was finally obtained. A fluorine-doped tin oxide (FTO) glass was then used as a carrier after being thoroughly washed by sonication treatment and subsequently dried. Two layers of TiO_2_ sol-based film was then deposited onto the FTO glass with a self-made film stretching machine at a stretching speed of 2 mm/s. Afterwards, the TiO_2_ sol-based film was thermally treated in a muffle furnace being heated to 500 °C at a heating rate of 2 °C/min, and kept at 500 °C for 1 h, followed by cooling to room temperature.

### 2.4. Preparation of TiO_2_ Nanotube-Based Film Electrodes by a Powder-Coating Method

TiO_2_ nanotubes (3.0 g) were placed in a mortar, followed by addition of 1 mL EtOH solution containing 10% diacetone and then ball-milling for 30 min. Afterwards, a mixture of DI water (4.5 mL), Triton X-100 (0.1 mL) and 30% (in weight, 0.9 g) of polyethylene glycol (M_W_ 20,000) was added, and then ball-milled for 30 min. The as-prepared mixed dispersion with a proper concentration was applied onto the above-prepared conducting FTO glass with two layers of TiO_2_ film by a powder-coating method. A scotch tape (with around 40 μm thickness) was attached vertically onto the two sides of the conducting FTO glass coated with the stretched film, and then the prepared TiO_2_ slurry was applied onto the conducting glass by rolling a glass bar. Rolling once in the same direction could result in the best film forming effect. The formed film with a size of 1 cm × 1 cm was vacuum-dried at 60 °C for 12 h, and then put into a muffle furnace, heated to 400 °C (at a heating rate of 2 °C/min), kept at that temperature for 1 h, and finally cooled to 80 °C for storage.

### 2.5. Sensitization of TiO_2_ Nanotubes with Bi_2_O_3_

**Figure 1:**
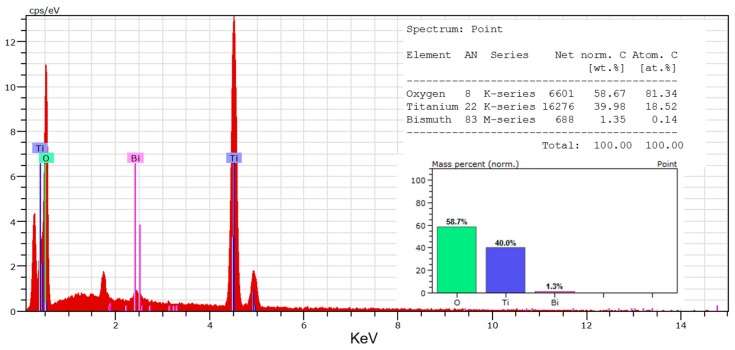
EDX spectrum of the typical sample, 0.05BiTNT, together with the quantification results presented as insets.

A precursor solution of Bi_2_O_3_ was first prepared as follows. Bi(NO_3_)_2_ solutions were prepared with molar concentrations of 0.01, 0.05, 0.15, 0.25, and 0.5 mol/L. The as-obtained TiO_2_ nanotube film was dipped into the Bi(NO_3_)_2_ solution and allowed to stand for 24 h, followed by washing with DI water and then drying under vacuum at 50 °C for 12 h. Afterwards, the treated film was placed into a muffle furnace which was heated to 400 °C at a heating rate of 2 °C/min, kept at that temperature for 5 h, and finally cooled naturally to 80 °C for storage. A series of samples were prepared, including pure TiO_2_ nanotube-based film (TNT), 0.01BiTNT, 0.05BiTNT, 0.15BiTNT, 0.25BiTNT, and 0.5BiTNT, where, for example, 0.01BiTNT represents that the product was prepared with the initial Bi(NO_3_)_2_ (a Bi_2_O_3_ precursor) solution of 0.01 mol/L. For clear demonstration of the sensitization effect of Bi_2_O_3_ on the TiO_2_ nanotubes, this study also investigated a simple mixture of TiO_2_ nanotubes and Bi_2_O_3_ particles being prepared by calcination of Bi(NO_3_)_2_ according to the above thermal treatment procedures. The weight percentage of Bi_2_O_3_ particles as used to mix with the TiO_2_ nanotubes was determined by quantification results of the energy-dispersive X-ray (EDX) spectrum for the optimized sample (Figure 1).

### 2.6. Characterizations

Transmission electron microscopy (TEM) observation was conducted on a JEOL JEM-1010 system (Tokyo, Japan). A JEM-2010HR system was used for the high-resolution TEM (HRTEM) analysis. Backscattered-electron (BSE) and EDX mapping images were captured on a tabletop microscope (TM3030Plus, Hitachi, Japan) being equipped with an EDX spectrometer (Quantax 70, Bruker, Germany). X-ray diffraction (XRD) analysis proceeded on a powder diffractometer (X’Pert-PRO, PANalytical, Philips, Amsterdam, The Netherlands); the test conditions were set as: Cu-Kα radiation, working voltage of 40 KV, working current of 40 mA, and scanning speed of 1.5°/min. The UV/Vis diffuse reflectance spectra (UV/Vis DRS) were captured from a UV-Vis-NIR spectrophotometer (LAMBDA 750, PerkinElmer, Norwalk, CT, USA), equipped with an integrating sphere. X-ray photoelectron spectroscopy (XPS) spectra were obtained from XSAM800-XPS equipment (Kratos, UK) with a Ma-Kα X ray source.

### 2.7. Photoelectric Conversion Properties of Bi_2_O_3_ Sensitized TiO_2_ Nanotube film 

**Figure 2:**
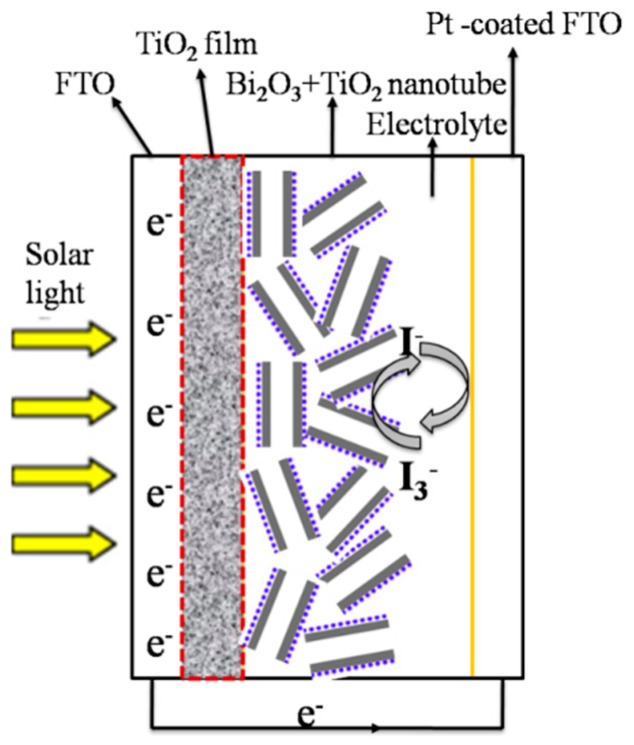
Schematic diagram of the Bi_2_O_3_ sensitized TiO_2_ nanotubes solar cell.

The dye-sensitized TiO_2_ nanotube-based anode film was replaced by Bi_2_O_3_-sensitized TiO_2_ nanotube film. Pt was then thermally deposited onto the conducting FTO glass as the other electrode. The two electrodes are assembled into a solar cell with a sandwiched structure, as displayed in Figure 2. Under xenon lamp irradiation conditions (300 W, AM 1.5, 100 mW/cm^2^, with the wavelengths below 400 nm filtered), IV curves are measured for the solar cell using electrochemical workstation. The incident-photon-to-current efficiency (IPCE) measurements were conducted using electrochemical workstation Zahner (PP211, CIMPS-pcs, Kronach, Germany) at the visible wavelengths of 400, 430, 450, 475, 500, 550, and 600 nm under xenon lamp irradiation conditions (300 W, AM 1.5, 100 mW/cm^2^).

A thermal deposition method was employed to fabricate the Pt counter electrode. A chloroplatinic acid EtOH solution with a proper concentration was first prepared at room temperature; specifically, 0.9 g of chloroplatinic acid was dissolved into 250 mL of EtOH. A dip-pulling method was then used to make a two-layer film onto a cleaned conductive glass with a self-made film-stretching machine. The thermal deposition was conducted at 390 °C (at a heating rate of 2 °C/min) and kept at the temperature for 0.5 h, followed by natural cooling.

The prepared electrolyte was an acetonitrile solution containing 0.1mol/L LiI, 0.05mol/L I_2_, and 0.5 mol/L Tert butyl pyridine.

## 3. Results and Discussion

**Figure 3:**
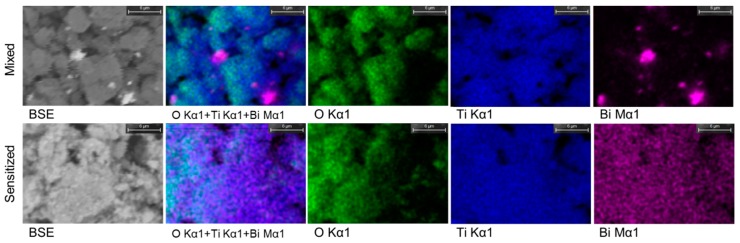
BSE and EDX mapping images of 0.05BiTNT (bottom row) and a simple mixture of TiO_2_ and Bi_2_O_3_ (top row).

This study first compares the microstructures of the typical sample, 0.05BiTNT, and a simple mixture of Bi_2_O_3_ and TiO_2_ nanotubes, with the same weight percentage of Bi_2_O_3_ as estimated by the quantification result of the EDX spectrum for 0.05BiTNT (Figure 1). From the EDX mapping images (Figure 3), considerably better distribution of element Bi can be found for 0.05BiTNT (bottom row) as compared to the simple mixture (top row) with the Bi_2_O_3_ particles showing apparently unsatisfactory adhesion interactions with the TiO_2_ matrix, which implies the importance and significance of the present sensitization method.

**Figure 4:**
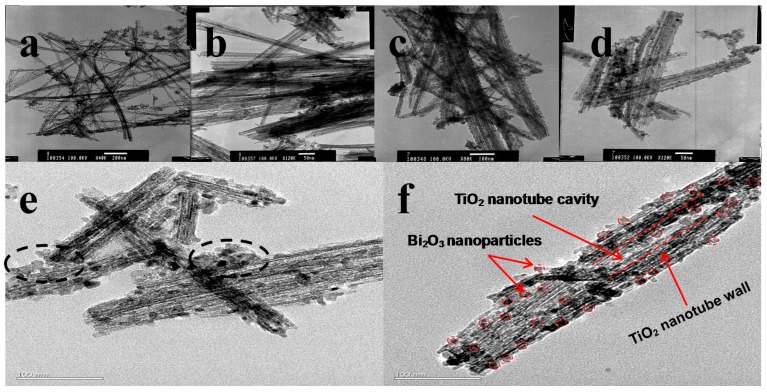
TEM and HRTEM images of the prepared samples. (**a**,**b**) Neat TiO_2_ nanotubes; (**c**–**f**) Bi_2_O_3_-sensitized TiO_2_ nanotubes. The detailed structural features are highlighted with red circles and lines in (**f**).

To further unravel the microstructure of the Bi_2_O_3_-sensitized TiO_2_ nanotubes, TEM and HRTEM are presented in Figure 4a–f. The TEM specimen was prepared by sampling from the coated FTO glass. Under low-magnification TEM (Figure 4a,b), neat TiO_2_ nanotubes show a large length-diameter ratio, with smooth tube walls. As shown in Figure 4c,d, the Bi_2_O_3_-sensitized TiO_2_ nanotube walls become coarsened, with the well-observed Bi_2_O_3_ nanoparticles coated on the walls. Under HRTEM (Figure 4e,f), it can be noted that the formed Bi_2_O_3_ nanoparticles are not fully deposited uniformly on the TiO_2_ nanotube walls, with a little amount of aggregates of Bi_2_O_3_ nanoparticles (highlighted by broken cycles). The average diameter of Bi_2_O_3_ nanoparticles can be calculated as around 10 nm.

**Figure 5:**
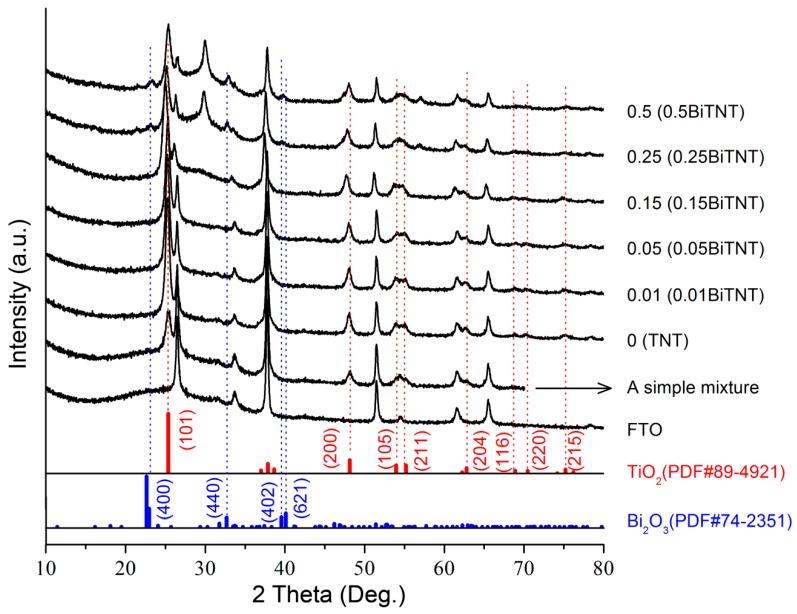
XRD patterns of a FTO glass and all the film samples deposited on the FTO glass, including a series of Bi_2_O_3_ sensitized TiO_2_ nanotubes and the simple mixture of TiO_2_ nanotubes and Bi_2_O_3_. The standard PDF cards of anatase TiO_2_ (No. 89-4921) and Bi_2_O_3_ (No. 74-2351) are also shown.

**Figure 6:**
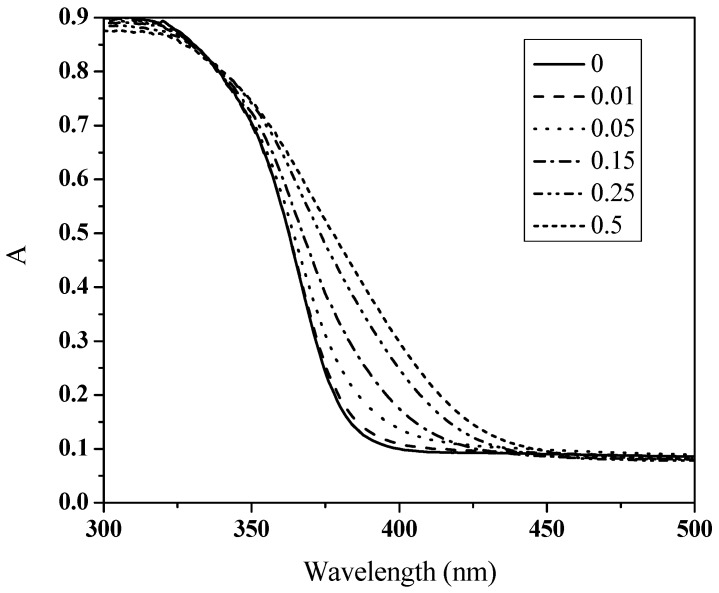
UV-Vis DRS of all the prepared samples.

The XRD patterns of the as-prepared coated FTO glass are shown in Figure 5. The characteristic peak assignable to FTO glass substrate can be noted. The anatase TiO_2_ crystal can also be well identified through the standard PDF cards of anatase (No. 89-4921) and its refection planes (highlighted by red dotted lines). As the concentration of the Bi_2_O_3_ precursor increases, the Bi_2_O_3_-related XRD bands gradually appear—as marked by blue dotted lines for showing the characteristic reflection planes of Bi_2_O_3_ (PDF#74-2351). Note that a low content of Bi_2_O_3_ for the sensitized samples such as 0.05BiTNT and the simple mixture of Bi_2_O_3_ and TiO_2_ causes the XRD peaks characteristic of Bi_2_O_3_ to be undetectable. From the UV/Vis spectra shown in Figure 6, the red shift of the absorption wavelength occurs for all the Bi_2_O_3_ sensitized TiO_2_ nanotube films. The higher the concentration of Bi_2_O_3_ precursor, the larger the red-shift extent, indicating the corresponding higher content of final formed Bi_2_O_3_ nanoparticles in the Bi_2_O_3_ sensitized TiO_2_ sample. This also indicates the successful sensitization of TiO_2_ by Bi_2_O_3_ [25].

**Figure 7:**
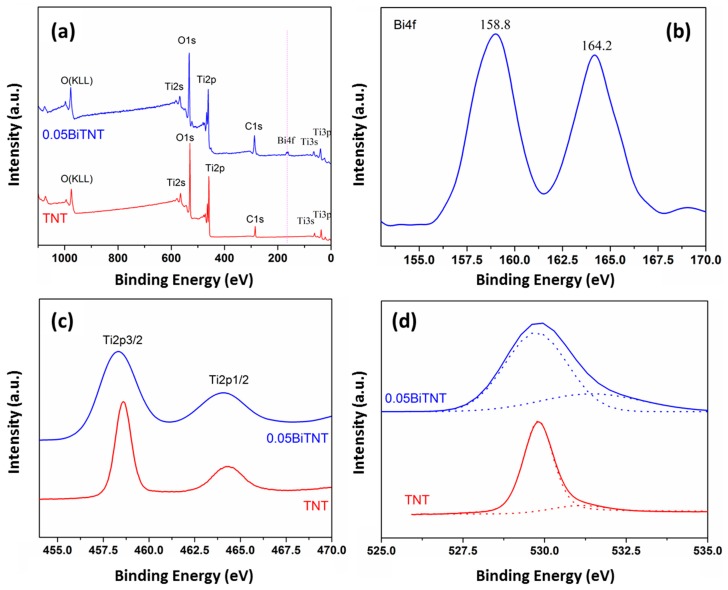
XPS studies of TNT and 0.05BiTNT; full XPS spectra (**a**); and high-resolution XPS spectra of Bi 4f (**b**); Ti 2p (**c**); and O 1s (**d**) regions.

To determine the elemental composition and obtain the corresponding chemical state information of the Bi_2_O_3_-sensitized TiO_2_ nanotube surface, XPS analysis is used to measure the typical sample TNT (as a control) and 0.05BiTNT. Figure 7a shows the full XPS spectra, verifying the elements Ti, O, Bi, and C (the element C most likely stems from the contaminant [26]) included in 0.05BiTNT. Due to the incorporation of Bi_2_O_3_, an additional signal from Bi 4f can be well observed in the full XPS spectrum for 0.05BiTNT, in contrast to that for TNT. The high-resolution XPS spectrum of Bi 4f is given in Figure 7b, and the two characteristic peaks at approximately 164.2 and 158.8 eV can be indexed to the Bi 4f_6/2_ and Bi 4f_7/2_ respectively [27]. The binding energy of Bi as for 0.05BiTNT is the same as that for pure Bi_2_O_3_, indicating that the element Bi of 0.05BiTNT presents in the chemical state of Bi_2_O_3_. The binding energy of Bi for 0.05BiTNT is in great agreement with those for reported pure Bi_2_O_3_ [28] and a Bi_2_O_3_–TiO_2_ composite [29], thus revealing that the element Bi of our 0.05BiTNT presents the chemical state of Bi_2_O_3_. This is also in good agreement with XRD analysis.

In the high-resolution XPS spectrum of Ti 2p (Figure 7c), the Ti 2p energy level is split into two energy levels, namely Ti 2p_1/2_ and Ti 2p_3/2_ as a result of spin-orbital coupling of electrons. The binding energy positions of Ti 2p_1/2_ and Ti 2p_3/2_ can be found at approximately 464.0 and 458.3 eV respectively, being basically the same as that reported elsewhere [30]. This also indicates that, both before and after the sensitization of TiO_2_ with Bi_2_O_3_, the oxidation state of the element Ti keeps the same, i.e., Ti^4+^, in the absence of Ti^3+^, and thus reveals the high purity of TiO_2_ [31]. The broadening of the XPS peaks after the Bi_2_O_3_ incorporation (0.05BiTNT vs. TNT) can be attributed to the electron transfer interactions between the matrix TiO_2_ and the sensitizer Bi_2_O_3_ [32]. The high-resolution XPS spectra of O 1s are shown in Figure 7d. Two peaks at 529.7 and 531.4 eV can be noticed by Gaussian fitting. The former can be indexed to the O of TiO_2_; the latter most likely results from Bi_2_O_3_, which may also be correlated to OH species from chemical absorption or absorbed water [25]. The lower binding energy of Bi–O is owing to the less negative changes of the covalent O of Bi_2_O_3_ as compared to that of the O of Ti–O [33]. To conclude, it is believed that the TiO_2_ nanotubes are effectively sensitized by Bi_2_O_3_ [25].

**Figure 8:**
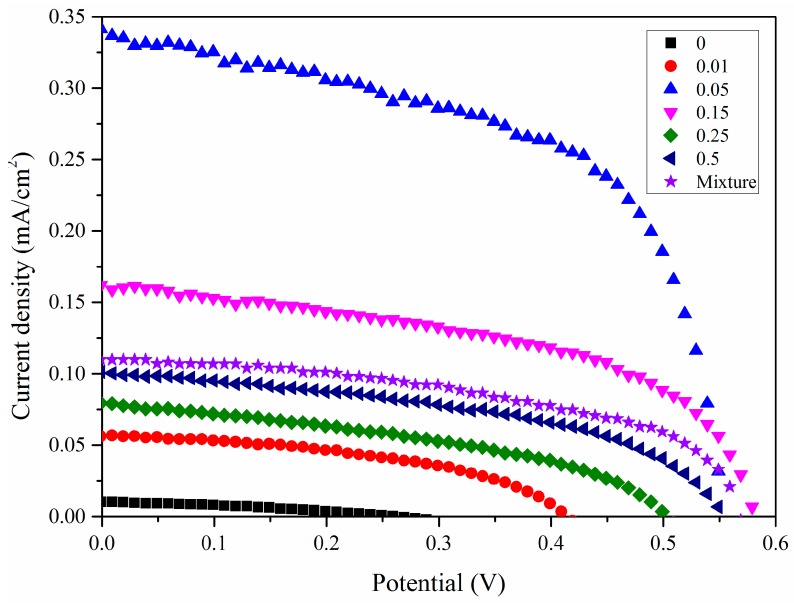
IV curves of a series of Bi_2_O_3_ sensitized TiO_2_-based solar cells, as well as a simple mixture of Bi_2_O_3_ and TiO_2_-based solar cell.

**Figure 9:**
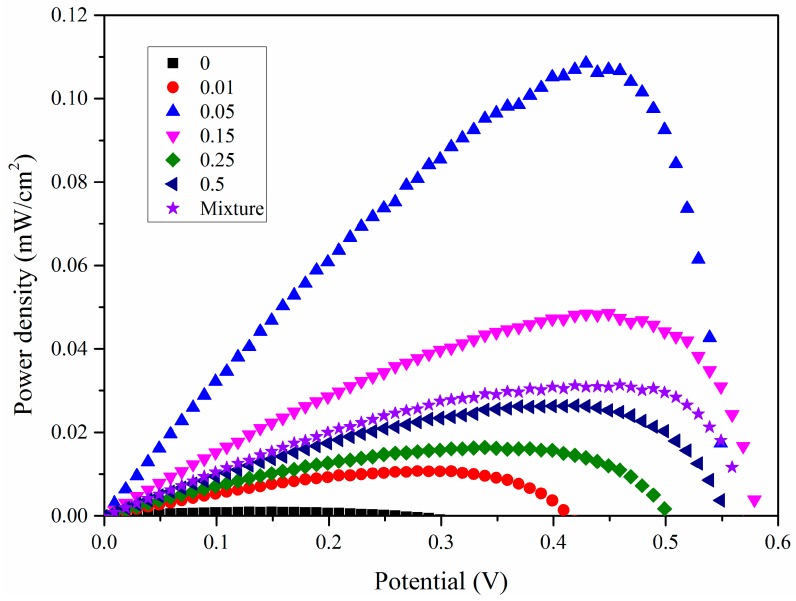
PV curves of a series of Bi_2_O_3_ sensitized TiO_2_-based solar cells, as well as a simple mixture of Bi_2_O_3_ and TiO_2_-based solar cell.

Figure 8 shows the IV curves (obtained for the assembled solar cell with the Bi_2_O_3_ sensitized TiO_2_ nanotube anode film). The corresponding PV curves of the Bi_2_O_3_ sensitized solar cell are presented in Figure 9. The photoelectric conversion efficiency of the DSSC is not directly proportional to the amount of sensitizer Bi_2_O_3_, and there exists an optimized content of Bi_2_O_3_. Our optimal content of the sensitizer Bi_2_O_3_ corresponds to the initial Bi_2_O_3_ precursor concentration of 0.05 mol/L. The lower content of Bi_2_O_3_ is most likely to cause insufficient utilization of visible light, while an excess amount of Bi_2_O_3_ exerts an adverse effect on the light absorption property of the light anode material, revealing that an optimized content of Bi_2_O_3_ exists. In DSSC, the optimal absorbed dye concentration lies in the monolayer adsorption of dye molecules onto the porous TiO_2_-based film electrode, which may be also applied to our case. To further prove the sensitization effect of Bi_2_O_3_ on TiO_2_ nanotubes, a simple mixture of Bi_2_O_3_ particles and TiO_2_ nanotubes is also investigated and compared with our optimized sample, 0.05BiTNT. The weight percentage of the Bi_2_O_3_ particles used for the mixing is estimated from the quantified result of the EDX spectrum for 0.05BiTNT (Figure 1). The simple mixture-based solar cell presents much lower photoelectric conversion efficiency as compared to the sensitized counterpart (0.05BiTNT), thereby indicating the significance of the sensitization effect as a result of uniform distribution of Bi_2_O_3_ and interfacial adhesion between the Bi_2_O_3_ and TiO_2_ nanotubes, as evidenced by EDX mapping images (Figure 3), and TEM and HRTEM images (Figure 4).

**Figure 10:**
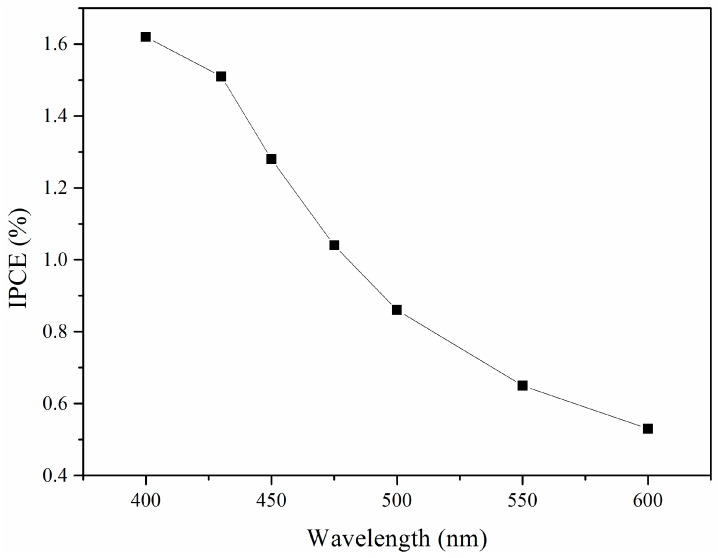
IPCE spectrum of the 0.05BiTNT-based solar cell, as captured at the incident wavelengths ranging from 400 to 600 nm.

From Table 1, it can be noted that, as the Bi_2_O_3_ content increases, the photoelectric conversion efficiency raises first and then exhibits a decreasing trend. The sample 0.05BiTNT presents the highest photoelectric conversion efficiency, followed by 0.15BiTNT > 0.5BiTNT > 0.25BiTNT > 0.01BiTNT > TNT. The un-sensitized TiO_2_ nanotubes (namely TNT)-assembled DSSC possesses a rather unsatisfactory photovoltaic property; this is because the present visible light source cannot excites the TiO_2_ nanotube film, with a wide energy band gap, to generate photocurrent and photovoltage, indicating the high significance of the present sensitization of TiO_2_ nanotubes with Bi_2_O_3_. Furthermore, a IPCE spectrum of the typical sample, 0.05BiTNT, shows a gradual lowering of IPCE with increasing incident wavelengths (starting from 400 to 600 nm), as presented in Figure 10.

## 4. Conclusions

In this study, a series of visible light-responsive Bi_2_O_3_ sensitized TiO_2_ nanotube anode films were prepared by combined hydrothermal reactions, dip-coating, and calcination. In terms of the photoelectric conversion efficiency of the assembled solar cells, the Bi_2_O_3_ precursor (Bi(NO_3_)_2_) concentration of 0.05 mol/L is estimated to be the optimal condition. The photoelectric conversion efficiency is not in direct proportion with the Bi_2_O_3_ content, and optimization needs to be performed. Similar to dye sensitization in DSSC, the monolayer adsorption of the sensitizer is most likely to be optimal for achieving high photoelectric conversion efficiency.

**Figure 11:**
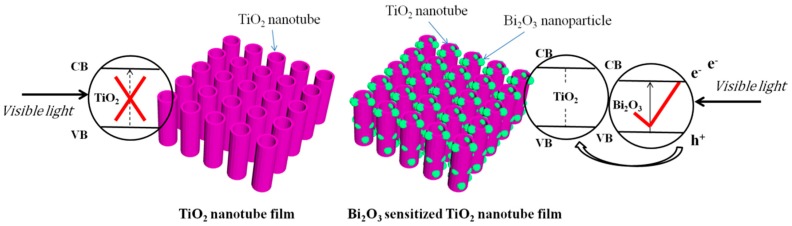
Photocatalysis mechanism of sensitizing TiO_2_ by Bi_2_O_3_.

As a new-type visible light-active photocatalyst, Bi_2_O_3_ is popular in the field of semiconductor materials. Bi_2_O_3_ has an energy band gap of 2.8 eV (*E*_CB_ = 0.33 eV, *E*_VB_ = 3.13 eV) at room temperature [34]. A single component photocatalyst, TiO_2_ nanotubes, shows a rather unsatisfactory visible light-driven photocatalytic property, but after sensitization with Bi_2_O_3_, its photoelectric conversion efficiency is largely improved, as schematically shown in Figure 11. Bi_2_O_3_ serves to absorb visible light energy to generate photoexcited electrons and holes, while TiO_2_ works to collect and transport the generated charge carriers [35]. Considering the different CB and VB positions of Bi_2_O_3_ and TiO_2_, a built-in electric field can be generated as a result of the energy level difference between Bi_2_O_3_ and TiO_2_, effectively separating the photo-generated electron-hole pairs and consequently improving the photocatalytic activity.

## Figures and Tables

**Table 1:** Photovoltaic characteristics of the Bi_2_O_3_ sensitized TiO_2_-based solar cells.

Electrodes	0	0.01	0.05	0.15	0.25	0.5
J_sc_ (mA/cm^2^)	0.0103	0.0666	0.341	0.161	0.0786	0.102
V_oc_ (mV)	269	414	664	681	603	664
P_max_ (mW/cm^2^)	9.41 × 10^−4^	0.0101	0.109	0.0490	0.0164	0.0267
